# Selected Morphological Characteristics, Lead Uptake and Phytochelatin Synthesis by Coffeeweed (*Sesbania exaltata* Raf.) Grown in Elevated Levels of Lead-Contaminated Soil

**DOI:** 10.3390/ijerph8062401

**Published:** 2011-06-23

**Authors:** Gloria Miller, Gregorio Begonia, Maria F. T. Begonia

**Affiliations:** Plant Physiology/Microbiology Laboratory, Department of Biology, College of Science, Engineering, and Technology, Jackson State University, P.O. Box 18540, 1400 Lynch Street, Jackson, MS 39217, USA

**Keywords:** coffeeweed, lead, morphology, phytochelatins, phytoextraction, *Sesbania*

## Abstract

Remediation of lead-contaminated soil is significant due to the inherent toxicity of lead (Pb), and the quantity of Pb discharged into the soil. One of the most cost-effective and environmentally sound technologies for the cleanup of metal-contaminated soils is through the use of plants. While much is known about the ecological evolution of metal tolerance in plants, the physiological, biochemical, and genetic mechanisms of tolerance is not well understood in the majority of resistant ecotypes such as the legume, *Sesbania exaltata* Raf. This study was therefore conducted to determine the morphological and physiological characteristics of *Sesbania* that had been grown in Pb-contaminated soil, and to assess phytochelatin synthesis as a way of elucidating its relative Pb tolerance. *Sesbania* plants were grown in the greenhouse and exposed to various levels of Pb: 0, 1000, and 2000 mg Pb/kg soil. Plants were harvested after 6, 8, and 10 weeks of growth and morphological characteristics (e.g., root and shoot biomass, root length, number of root nodules, shoot height, number of leaves, number of flowers, number and length of pods) were recorded. Generally, there were no statistical differences in morphological characteristics among the treatments. Further, no discernible phytotoxic symptoms, such as chlorosis, wilting, or necrotic lesions, in neither roots nor shoots were observed. We concluded that while *Sesbania* did not fit the model of a hyperaccumulator, the plant was, nonetheless, tolerant to elevated Pb levels. Our assessment for phytochelatin synthesis as a tolerance mechanism was inconclusive and further investigations of tolerance mechanisms are warranted.

## 1. Introduction

Soil contamination by heavy metals such as cadmium (Cd), copper (Cu), nickel (Ni), Zinc (Zn), and Pb has become a critical environmental concern due to potential chronic and acute ecological effects [1]. Remediation of contaminated soils by conventional engineering techniques can cost between $ 50 and $ 500 per ton and certain specialized techniques can exceed costs of $ 1000 per ton. With an acre of soil (to a 3-foot depth) weighing approximately 4500 tons, this could translate to a minimum cost of about a quarter million dollars per acre [2]. While the most common methods of managing the ever-growing number of contaminated sites are either the extremely costly processes of removal and burial, or simple isolation of the contaminated sites [3], there is an active effort to develop new, more cost-effective technologies using plants to remove toxic metals from soil (phytoremediation).

Phytoremediation is emerging as cost-effective and environmentally sound cleanup of metal-contaminated soils [4] in part because the costs of growing plants are minimal compared to those of soil removal and replacement [5]. Also, the disturbance to the environment is marginal and the secondary air- or water-borne waste is eliminated. A subset of phytoremediation known as phytoextraction is the use of plants to remove inorganic contaminants, primarily metals, from polluted soil. In this approach, plants capable of accumulating high levels of metals are grown in contaminated soil [6]. At maturity, metal-enriched aboveground biomass is harvested and a fraction of soil metal contamination removed [7–9]. The success of phytoextraction as an environmental cleanup technology, depends on several factors including the extent of soil contamination, metal availability for uptake into roots (bioavailability), and the plant’s ability to intercept, absorb, and accumulate metals in shoots [10].

Lead is not considered to be an essential element for plant growth and development. Lead inhibits growth, reduces photosynthesis (by inhibiting enzymes unique to photosynthesis), interferes with cell division and respiration, reduces water absorption and transpiration [11–13], accelerates abscission or defoliation and pigmentation, and reduces chlorophyll and adenosine triphosphate (ATP) synthesis [14]. Ultimately, the potential for phytoextraction depends on the interaction between soil, metal, and plant [7]. Crop selection, therefore, is an important factor for successful phytoremediation. *Sesbania exaltata* Raf., also commonly known as hemp *Sesbania* or coffeeweed, has been recognized as a possible phytoremediation species [15–19] because of its high biomass yield under elevated Pb levels, and its ability to translocate high amounts of Pb into its shoots when exposed to various concentrations of Pb and ethylenediaminetetraacetic acid (EDTA).

*Sesbania* occurs along ditches, roadsides, fields, disturbed sites, river banks and lake shores throughout much of the coastal plain region of Georgia, North and South Carolina, Florida, Virginia, Alabama, and Mississippi [20,21]*. Sesbania* is primarily a weed of agronomic crops, and can be quite competitive with cotton due to its rapid growth rate. It is a robust annual and can grow up to 4 meters tall with few or no branches. The plant has a taproot system. The stems are green without hairs, and may become woody with age. The flowers are yellow and may be streaked or spotted with purple [20,21]. Its distinctive curved seedpod, often tipped with a beak, contains 30 to 40 seeds [21]. The seed coats are from 59–63% impermeable, but can be made permeable by acid scarification or mechanical scarification [20].

Elemental toxicity in plants is a complex problem, the characteristics of which depend on the species, the element concentration and form, and the soil pH and composition. One of the objectives of this study was to assess the morphological and physiological characteristics of *Sesbania* that had been grown in Pb-contaminated soil.

Phytochelatin synthesis is reported to be the principal heavy-metal detoxifying factor in the plant kingdom [22] and is assumed to be involved in the accumulation, detoxification, and metabolism of metal ions such as Cd, Zn, Cu, Pb, and mercury (Hg), in plant cells [23,24]. Our second objective, therefore, was to assess phytochelatin synthesis as a way of elucidating the relative tolerance of *Sesbania* grown in Pb-contaminated soils.

## 2. Materials and Methods

In order to minimize discrepancies in the results that could arise from heterogeneous soil samples, a laboratory amended Pb-spiked soil sample was used throughout this experiment so that we could create a test sample with consistent lead concentration and speciation, soil composition, contamination process, and contamination period.

### 2.1. Soil Preparation

Delta top soil, a silt loam soil that is a member of the fine, Kaolinitic, thermic typic Kandiudult soils [25] and humus peat were allowed to air dry to approximately 1–3% moisture content for 3–4 days under greenhouse conditions. Top soil and peat were cleaned of debris using a 1 cm sieve. Soil was prepared by mixing sieved soil and peat in a 2:1 volumetric proportion. Representative samples of the prepared soil mixture were sent to Mississippi State University Soil Testing Laboratory, Mississippi State, MS to determine some physical and chemical characteristics of the soil.

Approximately 550 g of the dry, sieved Delta topsoil, peat mixture (2:1 v/v) were placed in a plastic zip lock bag and amended with either 0, 1000, or 2000 mg Pb/kg dry soil mixture using lead nitrate. Deionized distilled (dd) water was added to each bag of soil mixture to adjust the soil moisture content to approximately 30% field capacity. The bags of soil were left to equilibrate (age) on a laboratory bench in the greenhouse for six weeks. The bags were occasionally turned and mixed during the incubation period to ensure thorough mixing.

### 2.2. Sowing of Seeds

*Sesbania exaltata* Raf. seeds were purchased from Azlin Seed Co., Leland, MS. In order to soften the seed coat, *Sesbania* seeds were placed in a beaker that had been filled with dd water. The water was heated to 40 °C, the heat was turned off, and the seeds were left to soak in the water for 24 hr. To prepare the planting tubes, brown Wipe-All paper towels were folded and pushed to the bottom of a 656 mL Deepot tube (Stuewe and Sons, Inc., Corvallis, OR). The holes on the sides and at the bottom of the tubes were then wrapped with parafilm to prevent water from leaching from the bottom of the tube and to allow aeration at the root zone. Each Deepot tube was then filled with 550 g of the appropriate Pb-spiked soil mixture (0, 1000, 2000 mg Pb/kg dry soil). Six pre-soaked *Sesbania* seeds were planted in each tube and watered with 20 mL dd water.

### 2.3. Plant Establishment and Maintenance

*Sesbania* plants were irrigated every 2 days with 20 mL of either dd water or with a modified Hoagland’s nutrient solution. A 250-mL plastic cup was placed under each tube for leachate collection which also prevented cross contamination among treatments. Any leachate collected in each plastic cup was poured back into its corresponding tube. Periodically, these cups were rinsed with dd water and the resulting washing solutions were poured back into the respective tubes. The volume of water and/or nutrient solution ensured that soil moisture content was maintained at field capacity. Plants were maintained at a naturally-lit Jackson State University (JSU) greenhouse throughout the experimental period. Emerged seedlings were thinned to 2 plants per tube at 5 days after emergence. Additionally, for some designated treatments, ethylenediaminetetraacetic acid (EDTA) in a 1:1 ratio with the Pb, was applied as 100 mL aqueous solution 6 days before each harvest.

This experiment consisted of six treatments, arranged in a randomized complete block design (RCBD), with 2 plants per tube, and 4 replicates for each harvesting period (6, 8, and 10 weeks after seed emergence).

During harvest, shoots and roots were separated, and roots were washed with dd water to remove any adhering debris. The shoots and roots were then oven-dried in a Thelco convection oven (Precision Scientific Co., Chicago, IL) at approximately 75 °C for at least 48 h.

### 2.4. Measurement of Growth Parameters

Eight growth parameters were measured for each harvesting period: (1) Root lengths—after the roots were washed and towel blotted, root lengths were determined by measuring from the base of the stem (where the stem emerged into the soil) to the tip of the tap root. (2) Root nodules appearing along roots were counted. (3) Shoot heights were determined by measuring from the base of the stem to the shoot tip. (4) Leaves were counted from the bottom of the stem to the shoot tip (including abscised leaves). (5) Number of leaves that had abscised from the stem was counted. This was determined both by visual inspection and by feeling along the stem for leaf scars. (6) Flowers were counted. (7) Pods—the number of legumes (pods) produced by each plant was counted. (8) The length of each pod was measured.

### 2.5. Lead Extraction from Plant Tissues

Dried tissue samples were weighed and ground in a Wiley mill equipped with a 425 μm (40-mesh) screen. Lead contents were extracted using the U.S. Environmental Protection Agency USEPA) test method 3040B, with slight modifications [15]. Briefly, 40 mL of 50% aqueous nitric acid were added to a representative 200 mg sample of plant tissue. The acidified sample was heated to ∼35 °C, refluxed for 15 min without boiling and then allowed to cool. Another 10 mL of 50% aqueous nitric acid were added and the sample was again heated and refluxed for 30 min. The heated sample was allowed to cool, and then completely oxidized in 5 mL of concentrated nitric acid. The oxidized solution was then allowed to evaporate to approximately 5 mL without boiling. To initiate the peroxide reaction, 2 mL of dd water and 3 mL of 30% hydrogen peroxide (H_2_O_2_) were added to the concentrated digestate and then heated until effervescence subsided. Another 7 mL of 30% hydrogen peroxide were added continuously in 1 mL aliquots as the digestate was again heated. The digestate was heated until effervescence was minimal and its volume reduced to approximately 5 mL. After cooling, the final digestate was diluted to about 15 mL with dd water. The digestate was then filtered through a filter paper (Whatman No. 1) and the final volume was adjusted to 25 mL with dd water. Samples were analyzed for lead concentrations by Inductively Coupled Plasma-Optical Emission Spectrometry (ICP-OES; Perkin Elmer Optima 3300 DV).

### 2.6. Phytochelatin Assessment

In a corollary study, we assessed phytochelatin (PC) synthesis in coffeeweed after 8 weeks of growth in Pb-contaminated soil. Methods for soil preparation, sowing of seeds, plant establishment and maintenance, harvesting and measurement of growth parameters as described above were followed exactly.

*Sesbania* plants were grown in the greenhouse and harvested at 8 weeks after emergence. Phytochelatin extraction was performed using the procedures described by Keltjens [26]. Phytochelatin content was expressed as μmol/g dry weight. Total tissue glutathione was determined following the recycling method of Anderson [26,27] as briefly outlined below.

Two sets of plants were harvested and partitioned into shoots and roots. One set of plants was used for determination of shoot and root dry weights. Dry weights were measured after oven-drying fresh shoots and roots for at least 24 hr at ∼75 °C. The other set of plants was used for phytochelatin analyses. Roots were washed with dd water, blotted between paper towels, frozen with liquid nitrogen, and stored in a Thermo Electron Forma 86 ULT-80 Freezer (Thermo Electron Corp.) until they were removed for homogenization. The shoots were treated in a similar way, but without washing and blotting. Each sample was removed from the freezer and homogenized for 5 min in 10 mL of extraction solution with a mortar, pestle, and quartz sand. The extraction solution contained 5% (w/v) sulfosalicylic acid +6.3 mM diethylenetriaminepentaacetic acid (DTPA). The whole procedure was carried out under N_2_ gas to prevent oxidation.

The homogenate was cooled in ice and centrifuged for 15 min at 10,000 rpm in a Beckman Optima XL-100K Ultra Centrifuge at 4 °C. Five mL of the supernatant were used for phytochelatin analysis and the remaining 5 mL of supernatant were used for Pb analysis. Phytochelatin concentrations in the supernatant were determined indirectly by analyzing concentrations of total acid-soluble thiols (total SH) [28] and total glutathione (reduced glutathione [GSH], + oxidized glutathione [GSSG]). Total acid-soluble thiols were determined using Ellman’s reagent [5, 5′-dithio 2-nitrobenzoic acid (DTNB)]. The absorbance was read at 412 nm using a Single Beam Scanning UV/Visible Spectrophotometer. Concentrations were calculated using a molecular extinction coefficient of 13,600. Total glutathione was measured by the GSSG recycling method [27] with GSSG as a standard. Concentrations of Pb in the supernatant were determined by ICP-OES without further treatment of the samples. Three replicate treatments were analyzed.

### 2.7. Statistical Analysis

The greenhouse experiment consisted of 6 treatments, 2 plants per tube, and 4 replicate treatments for each of 3 harvesting periods (6, 8, and 10 weeks). Data were analyzed using Statistical Analysis System Version 9 (SAS V9). Treatment comparisons were done using Fisher’s Protected Least Significant Difference (LSD) test. A probability of less than 5% (*p* < 0.05) was considered to be statistically significant. This experiment was repeated two consecutive years.

## 3. Results and Discussion

For soil remediation initiatives, it is important to characterize both the chemical and physical parameters of the soil. Soil composition (e.g., nutrients, organic and inorganic materials), and soil mixture may all influence how the contaminant will behave. In general, the chemistry of metal interaction with soil matrix is central to the phytoremediation concept. While the soil used in this study was high in phosphorus, potassium and magnesium, these were, nonetheless, essential plant nutrients. Results of the soil analysis by Mississippi State University Soil Testing Laboratory showed that the parameters of the soil used in this study were well within limits for our objectives (Table 1).

The soil sample used in this experiment is a member of the fine Kaolinitic, thermic typic Kandiudult soils. These soils have a low, but not extremely low cation-exchange capacity [25]. In order to determine Pb mobility in this type of soil Miller *et al.* (2008), performed the classical sequential extraction by Tessier *et al.* (1979) on laboratory prepared Pb-spiked soil. The results of Miller’s sequential extraction revealed that the greater percentage of Pb was concentrated in the residual and exchangeable fractions of this soil type [29]. The residual fraction (fraction 5) can be thought of as what is left over after the first four fractions have been removed. It contains primary and secondary minerals which may hold trace metals within their crystal structure [30]. These metals are not expected to be released under normal environmental conditions.

The concentrations in plant parts depend both on intrinsic (genetic) and extrinsic (environmental) factors and vary greatly for different species and for different metals [31]. In our greenhouse experiment, we observed that both root (R Pb) and shoot (S Pb) Pb-tissue concentration generally increased with increasing levels of soil-Pb concentrations, with the highest level seen after 10 weeks of growth (Tables 2 and 3).

Three basic strategies by which higher plants can tolerate the presence of large amounts of metals in their environment have been reported: (1) exclusion, whereby transport of metals is restricted, and low, relatively constant, metal concentrations are maintained in a shoot over a wide range of soil concentrations; (2) accumulation, whereby metals are accumulated in nontoxic form in the upper plant parts at both high and low soil concentrations [31]; and (3) indicator, whereby shoot metal concentrations reflect those in the soil in a linear relationship [32,33].

### 3.1. Root and Shoot Pb-Tissue Concentration

**Figure 1.**
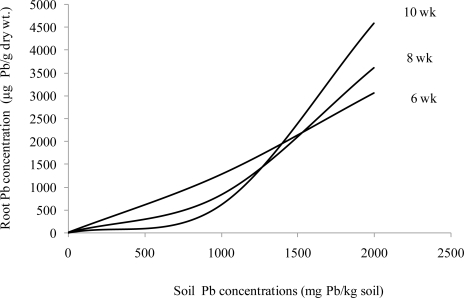
Effects of different Pb and EDTA treatments on root Pb concentrations (μg Pb/g dry wt.) of *Sesbania exaltata* Raf. at 6, 8, and 10 weeks after emergence.

**Figure 2.**
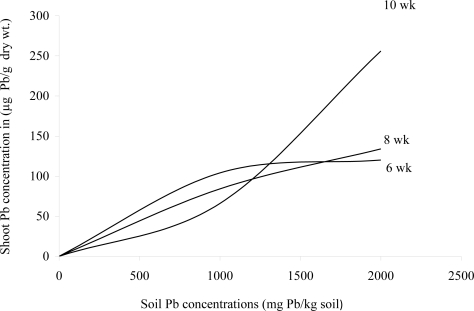
Effects of different Pb and EDTA treatments on shoot Pb concentrations (μg Pb/g dry wt) of *Sesbania* exaltata Raf. at 6, 8, and 10 weeks after emergence.

After 6 weeks of growth in 2000 mg Pb/kg soil, Pb concentrations in *Sesbania* root (Figure 1) and shoot (Figure 2) tissues more closely resembled the uptake curve of an indicator plant. After 10 weeks, however, the uptake resembled that of an excluder.

While both plants and animals respond to stress by using adaptations that help them evade, tolerate, or recover from stress [34], morphological plasticity allows plants to display different growth forms in response to different environmental conditions even without genetic change . As a result, organisms with the same genes often look different according to the environment [35]. Results of selected morphological characteristics of *Sesbania* roots and shoots grown in various concentrations of Pb are summarized in Tables 2 and Table 3.

### 3.2. Root and Shoot Biomass

The capacity of plants to remove contaminants from the soil is a function of biomass per unit area and concentration of the contaminant in the plants [36]. Generally, reduction of biomass production and nutritional quality is observed on crops grown in soils contaminated with moderate levels of heavy metal [11,37]. We observed that *Sesbania* roots biomass (Rb) did not differ significantly across the treatments (Table 2). Shoot biomass (Sb) followed the trend of root biomass for each harvesting period (Table 3).

### 3.3. Root Length

The root system of a plant constantly provides the stems and leaves with water and dissolved minerals. In order to accomplish this, roots must grow into new regions of the soil.

Although all plants take up metals to varying degrees from the substrates in which they are rooted [31], there is a general reduction of biomass production and nutritional quality on crops grown in soils contaminated with moderate levels of heavy metal [11]. For example, the height of plants or the size of their leaves may reflect the availability of water [35]. Root length and surface area, therefore, can be controlling variables for water and nutrient. Our results revealed that root lengths (Rl) were not significantly different among the treatments (Table 2). Generally, the longest roots were seen in treatments of 1000 mg Pb/kg soil, and the shortest roots were seen during week 10 in treatments of 2000 mg Pb/kg soil.

### 3.4. Root Nodules

The numbers of nodules (N) that formed along *Sesbania* roots were not significantly different among treatments for each harvesting period (6, 8, 10 weeks). However fewer (or no) nodules were formed along roots grown in treatments of 2000 mg Pb/kg soil (Table 2). Legumes are unique among higher plants in forming a symbiosis with nitrogen-fixing soil bacteria, collectively known as *Rhizobium* [38]. The physiological or developmental characteristics of legumes and actinorhizal plants that lead to predisposition for nitrogen-fixing root nodules are currently unknown.

### 3.5. Shoot Heights

The shoot heights (Sh) were not significantly different among the treatments during weeks 6, and 8, however by week 10, the heights (Sh) of plants grown in 2000 mg Pb/kg soil were significantly lower than Sh of plants grown in either 0 mg Pb/kg soil or 1000 mg Pb/kg soil (Table 3). Generally, shoot heights reflected the trend observed in root lengths.

### 3.6. Leaves

The numbers of leaves (L) produced by *Sesbania* shoots grown in Pb-contaminated soil were not significantly different among the treatments. The highest numbers of leaves were observed on plants harvested after 10 weeks of growth (Table 3).

### 3.7 Abscised

Leaf abscission (shedding of leaves) was not significantly different among the treatments (Table 3). Replacement of leaves in plants is a normal process. All leaves have a definite life span and are abscised following exposure to internal or environmental signals. Plants may also respond to environmental stimuli by altering their growth patterns [35], however, some stimuli induce senescence, which is a collective term for the processes accompanying aging that lead to the death of a plant or plant part [35]. We concluded that leaf abscission among treatments were normal since no other signs of metal toxicity such as chlorosis, necrotic lesions, yellowing of leaves, or wilting were observed.

### 3.8. Flowers

A flower (F) is a highly differentiated and specialized branch of the stem bearing modified leaves or flower parts. It is the site of sexual reproduction in *Sesbania* and other flowering plants, and is one of the plant’s most distinctive structures. The number of flowers (F) produced by *Sesbania* was not significantly different among the treatments (Table 3). The highest numbers were seen in week 8, in treatments of 0 mg Pb/kg soil.

### 3.9. Pods

A legume or pod is a dehiscent fruit which splits along its two seams and releases the enclosed seeds. It is a simple dry fruit that develops from a simple carpel and usually dehisces (opens along a seam) on two sides. A common name for this type of fruit is a “pod”. The numbers of pods (P) produced were not significantly different among the treatments. The highest numbers of pods was observed in plants after 6 weeks of growth.

### 3.10. Pod Lengths

Pod lengths (Pl) were not significantly different among the treatments. The longest pods (9.37 cm) were seen in week 8, in treatments of 2000 mg Pb/kg soil.

### 3.11. Phytochelatin (PC) Assessment

**Figure 3.**
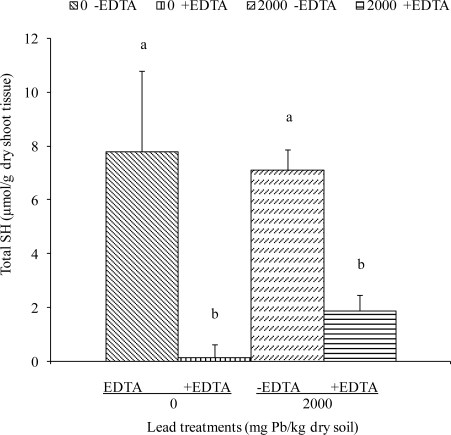
Total sulfhydryl (SH) nonprotein, acid soluble thiols in *Sesbania* shoots after 8 weeks of growth in Pb-contaminated soil. EDTA was added as an aqueous solution at 6 days before harvest. Values and error bars represent means and standard errors of 3 replicates. Treatments with common letters do not differ significantly from other treatments (Fisher’s LSD *p* < 0.05).

Although the measurements of total sulfhydryl (SH) nonprotein and acid soluble thiols are not specific for PC content, Grill and his colleagues [28] have estimated that in cells of *Rauvolfia serpentina*, at least 90% of the nonprotein SH groups accumulated in response to Cu^2+^ are accounted for by PCs. In the control (plants without Pb), the PC-SH concentration found in this study was not zero (Figure 3), a phenomenon that has been observed by other investigators [26,39]. There may be several reasons why this occurs, including the presence of microelements such as Cu and Zn that are present in all soils and hydroponic systems.

Our results revealed that the amounts of total sulfhydryl (SH) nonprotein, acid-soluble thiols accumulated in response to Pb/EDTA treatments were significantly higher in non-chelated plants (e.g., 7.91 and 7.11 μmol/g dry tissue for 0 mg Pb/kg and 2000 mg Pb/kg, respectively) as compared to plants treated with EDTA (e.g., 0.14 and 1.89 μmol/g dry tissue, respectively).

**Figure 4.**
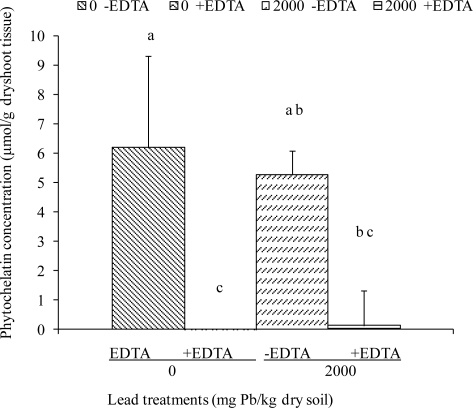
Phytochelatin concentrations of *Sesbania* shoots after 8 weeks of growth in Pb-contaminated soil. EDTA was added as an aqueous solution at 6 days before harvest. Values and error bars represent means and standard errors of 3 replicates. Treatments with common letters do not differ significantly (Fisher’s LSD, *p* < 0.05).

After adjusting for total glutathione (reduced glutathione [GSH], + oxidized glutathione [GSSG]), we found only negligible concentrations of PC in treatments of 2000 mg Pb/kg soil +EDTA (Figure 4).

These findings were in agreement with a study by Grill [22], wherein he and his colleagues reported that the chelation of a Cd^2+^ by EDTA as well as metal-free phytochelatin, instantaneously inhibited phytochelatin formation. Together, these results are in support of the hypothesis that PC synthesis proceeds until the metal ions are complexed and are no longer accessible to the synthase enzyme [22,24,40].

## 4. Conclusions

The results of this greenhouse study demonstrated that *Sesbania exaltata* Raf. was able to withstand elevated lead levels without displaying characteristic symptoms of metal toxicity such as chlorosis, necrotic lesions and wilting. We speculated that tolerance mechanisms may have enabled *Sesbania* to live (and grow) under elevated lead levels, although our assessment for phytochelatin synthesis as a tolerance mechanism was inconclusive.

Ultimately field phytoremediation requires an integrated approach, involving (1) type and depth of contamination, (2) irrigation and drainage water management strategies, (3) chemical and mineralization transformations within the soil, (4) pest management, (5) harvesting techniques, (6) end-use and disposal of biomass economic feasibility, (7) social acceptance [41], and (8) the initial selection of crops and crop rotation schemes. Our results support previous studies that show *Sesbania* to be a Pb tolerant plant and that with biotechnology may be a suitable species for phytoremediation.

## Figures and Tables

**Table 1.** Physical and chemical characteristics of the soil.

Characteristic	Extractable levels (kg/ha)
Soil acidity (pH)	6.3
Phosphorus	146*
Potassium	337*
Calcium	5081
Magnesium	813**
Zinc	4.7*
Sodium	180
CEC	17.6
% Clay	7.50
% Silt	80.08
% Sand	12.4

* High;

** Very High.

**Table 2.** The effects of Pb on growth parameters of *Sesbania* roots at 6, 8, and 10 weeks after emergence.

Mg Pb/Kg soil *EDTA	**Parameters after each harvest**			
	Six weeks of growth			
	RRRb	Rl	N RPb	
0	28.25 ^c^	10.95 ^ab^	4.38 ^a^	0 ^c^
0 *	31.62 ^c^	9.68 ^b^	5.38 ^a^	0 ^c^
1000	61.75 ^a^	16.51 ^a^	4.00 ^a^	1030.0 ^abc^
1000 *	52.25 ^ab^	16.03 ^a^	6.00 ^a^	1276.0 ^abc^
2000	38.62 ^bc^	13.65 ^ab^	0 ^a^	3676.0 ^a^
2000 *	32.37 ^bc^	12.70 ^ab^	1.00 ^a^	3068.0 ^ab^
	Eight weeks of growth			
	Rb	Rl	N	RPb
0	51.38 ^a^	11.59 ^bc^	6.50 ^a^	0 ^b^
0 *	36.08 ^a^	10.16 ^c^	5.12 ^a^	0 ^b^
1000	43.40 ^a^	14.60 ^ab^	4.83 ^a^	683.3 ^b^
1000 *	51.68 ^a^	16.72 ^a^	1.83 ^a^	822.2 ^b^
2000	47.59 ^a^	14.76 ^ab^	0^a^	3645.9 ^a^
2000 *	58.28 ^a^	11.59 ^bc^	0^a^	3609.5 ^a^
	Ten weeks of growth			
	Rb	Rl	N	RPb
0	54.25 ^a^	10.95 ^a^	6.75 ^a^	0^c^
0 *	54.38 ^a^	9.52 ^a^	6.33 ^a^	0^c^
1000	49.88 ^a^	11.90 ^a^	10.00 ^a^	1099.7 ^bc^
1000 *	52.00 ^a^	10.64 ^a^	1.00 ^a^	608.5 ^bc^
2000	32.50 ^a^	9.52 ^a^	0.65 ^a^	2417.5 ^b^
2000 *	40.75 ^a^	9.20 ^a^	0^a^	4582.6 ^a^

Data were calculated from four replicated treatments.

* indicates that EDTA was added as an aqueous solution 6 days prior to harvest. Plants were harvested after growing in Pb-spiked soil for 6, 8, and 10 weeks. In each column, within each harvesting period, the values with different letters (a,b,and c,) are significantly different according to ANOVA and Fisher’s test (*p* < 0.05). Rb = root biomass, Rl = root length, N = nodules, R Pb = Root tissue Pb concentration.

**Table 3.** The effects of Pb on growth parameters of *Sesbania* shoots at 6, 8, and 10 weeks after emergence.

mg Pb/kg soil *EDTA	**Parameters after each harvest**							
	Six weeks of growth							
	Sb	Sh	L	A	F	P	Pl	SPb
0	168.62 ^b^	28.57 ^b^	11.63^a^	5.25 ^a^	0.67 ^a^	0.87 ^a^	5.93 ^a^	0^c^
0 *	190.75 ^ab^	27.62 ^ab^	12.13 ^a^	6.37 ^a^	0.75 ^a^	0.87 ^a^	6.67 ^a^	0^c^
1000	285.62 ^a^	34.13 ^a^	13.25 ^a^	7.50 ^a^	0.87 ^a^	1.00 ^a^	6.67 ^a^	85.35 ^b^
1000 *	215.62 ^ab^	28.89 ^a^	12.00 ^a^	4.87 ^a^	1.00 ^a^	1.00 ^a^	7.30 ^a^	103.95 ^ab^
2000	214.62 ^ab^	29.69 ^a^	13.00 ^a^	6.25 ^a^	0.75 ^a^	0.87 ^a^	7.93 ^a^	142.83 ^a^
2000 *	211.87 ^ab^	26.19 ^a^	12.12 ^a^	7.25 ^a^	0.83 ^a^	0.87 ^a^	7.19 ^a^	119.82 ^ab^
	Eight weeks of growth							
	Sb	Sh	L	A	F	P	Pl	SPb
0	394.37 ^a^	42.55 ^a^	12.75 ^b^	5.87 ^a^	1.87 ^a^	0.67 ^a^	6.82 ^ab^	0^c^
0 *	239.62 ^a^	33.97 ^ab^	13.12 ^b^	5.37 ^a^	1.87 ^a^	0.62 ^a^	8.04 ^b^	0^c^
1000	303.25 ^a^	37.15 ^ab^	13.62 ^b^	6.37 ^a^	1.33 ^a^	0.83 ^a^	6.77 ^ab^	44.64 ^bc^
1000 *	368.37 ^a^	41.43 ^ab^	16.50 ^a^	5.25 ^a^	1.12 ^a^	0.67 ^a^	4.76 ^ab^	83.75 ^ab^
2000	341.87 ^a^	32.23 ^b^	11.37 ^b^	5.00 ^a^	1.25 ^a^	0.75 ^a^	7.14 ^ab^	131.77 ^a^
2000 *	385.87 ^a^	36.83 ^ab^	13.50 ^b^	6.37 ^a^	1.50 ^a^	0.87 ^a^	9.37 ^a^	134.28 ^a^
	Ten weeks of growth							
	Sb	Sh	L	A	F	P	Pl	SPb
0	340.12 ^abc^	51.12 ^a^	14.75 ^ab^	7.75 ^a^	1.50 ^a^	0.50 ^a^	3.39 ^a^	0^c^
0 *	482.00 ^a^	50.96 ^a^	15.12 ^ab^	7.62 ^a^	1.75 ^a^	0.50 ^a^	4.02 ^a^	0^c^
1000	439.37 ^ab^	58.04 ^a^	16.50 ^a^	8.75 ^a^	1.67 ^a^	0.50 ^a^	3.17 ^a^	78.37 ^bc^
1000 *	420.50 ^abc^	56.20 ^a^	16.62 ^a^	8.25 ^a^	1.50 ^a^	0.50 ^a^	4.28 ^a^	65.72 ^bc^
2000	251.75 ^bc^	31.91 ^b^	12.37 ^b^	6.62 ^a^	1.50 ^a^	0.67 ^a^	4.66 ^a^	125.56 ^b^
2000 *	244.12 ^c^	35.24 ^b^	11.87 ^b^	6.50 ^a^	0.67 ^a^	0.67 ^a^	4.02 ^a^	256.26 ^a^

Data were calculated from four replicated treatments.

* indicates that EDTA was added as an aqueous solution 6 days prior to harvest. Plants were harvested after growing in Pb-spiked soil for 6, 8, and 10 weeks. In each column, within each harvesting period, the values with different letters (a,b,and c,) are significantly different according to ANOVA and Fisher’s test (*p* < 0.05). Sb = shoot biomass, Sh = shoot height (cm), L = leaves, A = abscised leaves, F = flowers, P = pods, Pl = pod length (cm), S Pb = Shoot tissue Pb concentration.
